# Recovery time from severe acute malnutrition and associated factors among under-5 children in Yekatit 12 Hospital, Addis Ababa, Ethiopia: a retrospective cohort study

**DOI:** 10.4178/epih.e2020003

**Published:** 2020-02-02

**Authors:** Mekonen Adimasu, Girum Sebsibie, Fikrtemariam Abebe, Getaneh Baye, Kerebih Abere

**Affiliations:** 1 School of Nursing, Addis Ababa University College of Health Sciences, Addis Ababa, Ethiopia; 2 Debre Berhan University College of Medicine, Debre-Berhan, Ethiopia

**Keywords:** Recovery time, Severe acute malnutrition, Children, Under-5 children, Ethiopia

## Abstract

**OBJECTIVES:**

Recovery time from severe acute malnutrition (SAM) is often a neglected topic despite its clinical impact. Although a few studies have examined nutritional recovery time, the length of hospitalization in those studies varied greatly. Therefore, the aim of this study was to determine the recovery time from SAM and to identify predictors of length of hospitalization among under-5 children.

**METHODS:**

A retrospective cohort study was conducted among 423 under-5 children with SAM who had been admitted to Yekatit 12 Hospital. Kaplan-Meier analysis was used to estimate time to nutritional recovery, and Cox proportional hazard regression analysis was performed to determine independent predictors.

**RESULTS:**

The nutritional recovery rate was 81.3%, and the median recovery time was 15.00 days (95% confidence interval [CI], 13.61 to 16.39). Age, daily weight gain per kilogram of body weight, vaccination status, and the existence of at least 1 comorbidity (e.g., pneumonia, stunting, shock, and deworming) were found to be significant independent predictors of nutritional recovery time. The adjusted hazard ratio (aHR) for nutritional recovery decreased by 1.9% for every 1-month increase in child age (aHR, 0.98; 95% CI, 0.97 to 0.99).

**CONCLUSIONS:**

The overall nutritional recovery time in this study was within the Sphere standards. However, approximately 13.0% of children stayed in the hospital for more than 28.00 days, which is an unacceptably large proportion. Daily weight gain of ≥8 g/kg, full vaccination, and deworming with albendazole or mebendazole reduced nutritional recovery time. Conversely, older age, pneumonia, stunting, and shock increased nutritional recovery time.

## INTRODUCTION

Malnutrition is a multifaceted phenomenon. It encompasses overnutrition, which manifests as overweight or obesity; undernutrition, which includes acute and chronic malnutrition; and micronutrient deficiencies. In developing countries, undernutrition is associated with > 50% of deaths caused by infectious disease. Worldwide, there were around 60 million and 20 million children with moderate acute malnutrition and severe acute malnutrition (SAM), respectively, in 2013 [1,2].

SAM is defined as a weight-for-height (WFH) measurement below 70% or a WFH z-score below 3 standard deviations from the mean. It can also be defined as the presence of bilateral pitting edema of nutritional origin or a mid-upper arm circumference (MUAC) of less than 11 cm in children aged 1-5 years [3].SAM is defined as a weight-for-height (WFH) measurement below 70% or a WFH z-score below 3 standard deviations from the mean. It can also be defined as the presence of bilateral pitting edema of nutritional origin or a mid-upper arm circumference (MUAC) of less than 11 cm in children aged 1-5 years [3].

Devastatingly, the number of children with SAM is still growing globally. SAM is the third most common contributing factor to the deaths of under-5 children worldwide. According to the World Health Organization, SAM causes 1 million deaths annually via increased susceptibility to death from severe infection [4]. Globally, children suffering from SAM have a risk of death that is 5 times to 20 times greater than that of well-nourished children. SAM can directly cause death, or it can indirectly increase the fatality rate among children suffering from diarrhea and pneumonia [3]. Although SAM is considered a minor issue in developed countries, it poses a major problem in Asia and Africa [5].

Based on the 2016 Ethiopian Demographic Health Survey (EDHS) report, Ethiopia has the region’s highest rate of acute malnutrition, with 3% of under-5 children identified as severely wasted. The prevalence of SAM showed little change from the 2011 EDHS data [6,7]. Additionally, Ethiopia has one of the highest under-5 child mortality rates in the region, with malnutrition underlying 28% of all child deaths [8].

A longer duration of hospitalization is associated with a higher risk of local and systemic infection. A cohort study in Malawi examined found significant correlations between markers of infection and length of hospitalization (p< 0.001) [9]. Another longitudinal study found that hospital-acquired infection was associated with prolonged hospitalization and a higher risk of in-hospital mortality [10].

Inpatient treatment programs for children with SAM have serious economic disadvantages. For instance, a cost analysis study that compared the costs of outpatient and inpatient treatment in Ethiopia reported the mean cost per child treated to be US$284.56 in an inpatient facility and US$134.88 in an outpatient center. A similar study in West Africa reported that the outpatient and inpatient treatment costs per child were €75.50 and €134.57, respectively [11,12].

Moreover, when mothers or caregivers stay with hospitalized children for a long period, the whole family loses labor and economic productivity, and this also poses challenges for other children at home [13].

Another devastating outcome of a longer duration of SAM is the long-term impact of SAM on nervous system development. Studies of humans and animals have revealed that prolonged starvation in infants and children results in clinical neurological deficits, including learning deficits and behavioral problems [14,15].

The impact of long-term hospitalization of under-5 children extends beyond the children themselves. A cohort study in Italy found that hospitalization of children for over 16 days produced a significant increase in stress among mothers or caregivers compared to shorter hospitalizations [16]. Several retrospective cohort studies have been conducted in different regions of Ethiopia to investigate recovery time of under-5 children from SAM. The estimated average recovery rate ranged from 43.6% at Ayder Referral Hospital to 83.0% at Wolisso St. Luke Catholic Hospital with a median recovery time ranging from 10.00 days at Dilchora Referral Hospital to 21.56 days at Ayder Referral Hospital [17-21]. The main predictors of recovery time have been found to be age [21- 24]; sex [18,25]; vaccination status [18]; type of malnutrition [19, 26]; baseline anthropometric measurements [18,27]; comorbidities, such as human immunodeficiency virus [18,22], pneumonia [19], anemia [22], dehydration [28] and rickets [19]; and treatments and supplements, such as antibiotics and folic acid [22].

However, the determinant factors of recovery time from SAM have not been explored in the region analyzed in the current study, specifically Yekatit 12 Hospital in Addis Ababa. Addis Ababa is unique socioeconomically and is inhabited by a large population of street children and orphans. Although studies have been conducted on nutritional recovery time in Ethiopia, some important variables, such as stunting and the WFH z-score at admission, were not assessed, and the length of hospitalization varied greatly in those studies [18,20,22,29,30]. Hence, the present study was designed to determine the recovery time from SAM and to identify predictors of length of hospitalization among under-5 children at Yekatit 12 Hospital in Addis Ababa City Administration, Ethiopia.

## MATERIALS AND METHODS

### Study design, area, and period

A facility-based retrospective cohort study was conducted at Yekatit 12 Hospital in Addis Ababa between February 2019 and March 2019. Addis Ababa is the capital city of Ethiopia and the seat of both the African Union and the Economic Commission for Africa. It had an estimated population of over 7 million people as of the end of 2019 [31].

Yekatit 12 Hospital is an organized health facility with 12 isolated beds. The facility employs well-equipped health care workers trained in the management of SAM using a standardized management protocol that was updated by the Federal Ministry of Health in 2007 [32]. The hospital also has an isolated therapeutic feeding unit (TFU), assigned nurses, and necessary equipment for the preparation of formula milk in the pediatric ward.

### Source and study population

All under-5 children who were admitted to Yekatit 12 Hospital for management of SAM between January 1, 2016 and December 30, 2018 were used as the source population. The study population comprised randomly selected under-5 children from the source population who were admitted to and treated at Yekatit 12 Hospital.

### Sample size determination

The sample size in this study was determined using a single-proportion formula:

$$n=\frac{{\left(Z_{\alpha /2}\right)}^{2}p\left(1-p\right)}{d^{2}}$$

Here, p is an estimate of the recovery rate (assumed to be 52%). This value was obtained from a retrospective study in Bahir Dar, since that study took place near Addis Ababa and was conducted recently (2018), and since the choice of this value resulted in a larger sample size [18].

n= 1.96^2^ × 0.52(0.48)/0.05^2^ = 384

After addition of 10% to the sample size to account for missing and incomplete data, the final sample size was 423 under-5 children with SAM.

### Sampling procedures

Three consecutive years (2016, 2017, and 2018) were purposively selected for record reviews because they provided the most recent available information about the problem under investigation at the selected institution.

All SAM cases were obtained from the TFU register book. There was no cyclic pattern in the ordering of the subjects on the list. Hence, systematic sampling was employed to select a sufficient number of samples starting from the most recent month and going backwards, based on the sequence of medical card numbers. The total number of under-5 SAM admissions during the 3-year period was 1,050. The total sample size for each year was allocated proportionally by calculating the interval from the total population (N) of the sampling frame (2016, N= 254; 2017, N= 356; 2018, N= 440) and the sample size n (k= N/n). The interval (k= 2) was similar for each year. The first number was selected randomly.

### Methods of data collection

A structured data abstraction form was used for data collection. The data abstraction form was adopted from the Ethiopian protocol for the management of SAM [32] and from previous studies [18,22,33,34]. Data regarding baseline characteristics such as socio-demographic status, immunization status, and baseline anthropometric measurements; type of malnutrition; comorbidities; routine medications, supplements and therapeutic feedings; and recovery time were retrieved from the clinical records of patients by trained health professionals. Moreover, the data abstraction form was pre-tested in 5% of the sample at Zewditu Memorial Hospital.

### Data collection procedure

The data collectors were 2 master’s degree students and 2 bachelor of science nurses. One supervisor was needed to manage the overall data collection process.

Medical card reviewers were provided with 2 days of training. A separate orientation was given to the supervisor regarding how to oversee the data collectors and how to check the completion of the data abstraction forms.

### Dependent and independent variables

The outcome variable was the recovery time from SAM, and independent variables included socio-demographic variables, type of malnutrition, baseline anthropometric measurements, immunization status, comorbid medical conditions, treatments, supplements, and therapeutic feeding.

### Operational definitions

In this study, recovery time referred to the number of days from admission until a child’s recovery from SAM. Recovered children were those who became free from medical complications and edema and achieved and maintained a sufficient MUAC (≥ 12.5 cm) and WFH (≥ 85%); these children were described as cured or recovered on their medical charts [35].

Censored children were children whose deaths were recorded, whose cases involved actions taken against medical advice, or who were lost during treatment with unknown status. A comorbidity was defined as a medical problem present in addition to SAM before or after admission. Finally, baseline anthropometric measurements (e.g., MUAC, weight, and height values) were those recorded at the time of admission.

### Data quality control

To ensure data quality, a pre-test was conducted on 5% of the sample. Any error found in the data abstraction format during the pre-test process was corrected. Then, the actual data were collected under close supervision. After collection, the data were carefully entered, cleaned, coded, and analyzed with SPSS version 25 (IBM Corp., Armonk, NY, USA). Afterward, the investigator cleaned the data in an orderly fashion by first sorting each variable in ascending order to check for unexpected cases.

### Methods of data analysis

The data were checked, coded, and entered into EpiData version 4.2 (EpiData Association, Odense, Denmark) and exported to SPSS for analysis. Graphs and frequency tables were used to report the descriptive data.

Recovery time from SAM was estimated using the Kaplan-Meier method. Then, bivariate Cox regression analysis was performed for each predictor variable regarding time to recovery. An adjusted hazard ratio (aHR) with 95% confidence interval (CI) was used to identify predictor variables of recovery at 4 weeks, and p-values < 0.05 were considered to indicate statistical significance.

### Ethics statement

Ethical clearance was obtained from the Institutional Review Board of Addis Ababa University, College of Health Sciences, School of Nursing and Midwifery. Permission was obtained from the board of Yekatit 12 Hospital. The privacy and confidentiality of study participants were maintained by ensuring that the data abstraction form was anonymous and by protecting our personal computers with strong passwords.

## RESULTS

**Figure 1.**
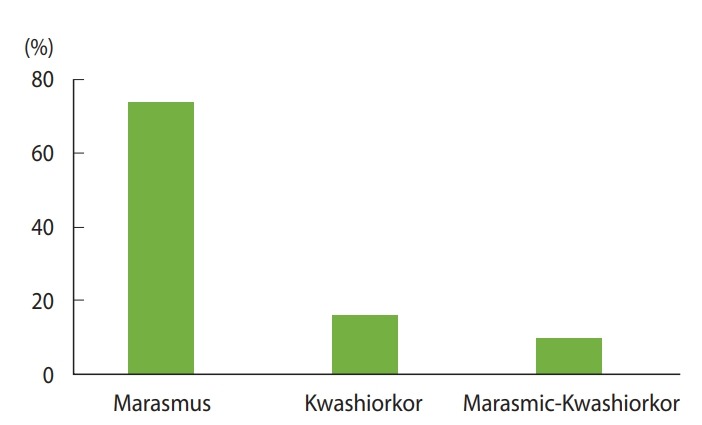
Distribution of type of malnutrition among admitted under-5 with severe acute malnutrition children in therapeutic feeding unit of Yekatit 12 Hospital from January 1, 2016 to December 30, 2018.

**Figure 2.**
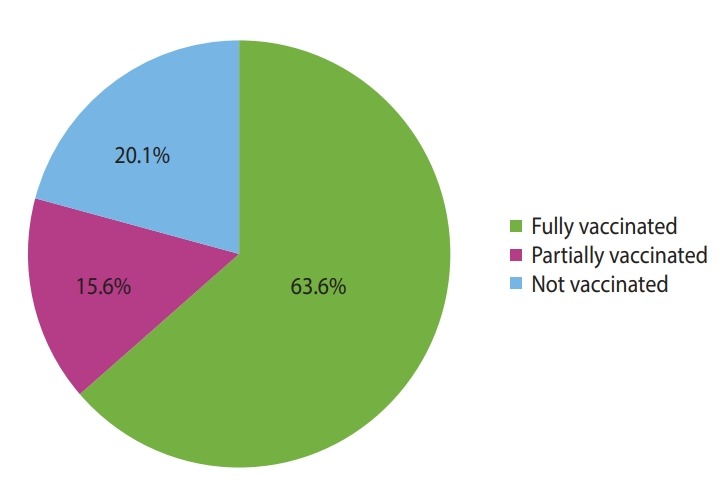
Vaccination for age status of under-5 children with severe acute malnutrition admitted in the therapeutic feeding unit of Yekatit 12 Hospital, Addis Ababa, Ethiopia from January 1, 2016 to December 30, 2018.

The study included records of 423 under-5 children with the diagnosis of SAM admitted over 3 consecutive years (2016, 2017, and 2018) to the TFU of Yekatit 12 Hospital in Addis Ababa, Ethiopia. Of the 423 children in the cohort, 241 (57.0%) were boys, and more than two-thirds (n= 289; 68.3%) of children were urban residents. The ages of the children ranged from 1 month to 59 months, with a median age of 11 months, and the majority of the children (n= 341; 80.6%) were younger than 24 months. Overall, 313 (74.0%) of the under-5 children admitted to the TFU had marasmus, and 69 (16.3%) had kwashiorkor. Similarly, 269 (63.6%) of the children were vaccinated fully and 66 (15.6%) of them were vaccinated partially for their age (Figures 1 and 2).

Among all under-5 children included in this study, 405 (95.7%) had at least 1 comorbid disease. The most common medical comorbidities were diarrheal diseases (53.0%), anemia (42.8%), pneumonia (42.3%), and fever (33.1%) (Table 1).

Of the 423 children whose medication records were selected for review, the most prescribed routine medications were intravenous antibiotics (n= 378; 89.4%) and oral antibiotics (n= 205; 48.5%). Regarding deworming, only 85 (20.1%) children were eligible (≥ 2 years) to take albendazole or mebendazole (Table 2).

### Kaplan-Meier survival estimates for severe acute malnutrition recovery time

**Figure 3.**
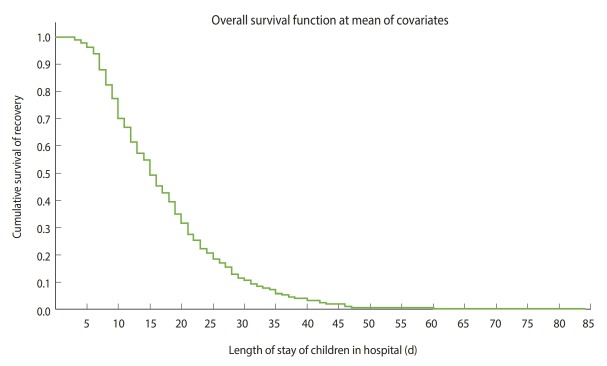
Shows overall Kaplan-Meier estimation of survival time to recover from severe acute malnutrition among under-5 children managed at Yekatit 12 Hospital from January 1, 2016 to December 30, 2018.

The cumulative likelihood of recovery at the end of the first, second, third, and fourth weeks was 12.0%, 45.0%, 73.0%, and 87.0%, respectively. Additionally, 13.0% of children stayed longer than 28.00 days, and approximately 3.0% of children stayed for more than 42.00 days (Figure 3).

The nutritional recovery rate was 5.29 (95% CI, 4.76 to 5.88) per 100 person-day observations, and the median nutritional recovery time of the entire cohort was 15.00 days (95% CI, 13.61 to 16.39). The median recovery time decreased from 16.00 days (95% CI, 13.88 to 18.12) in 2016 to 15.00 days in both 2017 (95% CI, 12.90 to 17.10) and 2018 (95% CI, 13.07 to 16.92).

Further analysis of the median survival time showed that significant differences in the median nutritional recovery time (p< 0.05) were associated with different predictors (Table 3).

### Factors associated with recovery time from severe acute malnutrition

Thirty-nine independent variables were analyzed in the Cox proportional hazard analysis along with the dependent variable. Twenty-four variables with p-values< 0.25 in the bivariate Cox analysis were entered into the multivariate Cox proportional hazard regression analysis. However, only 7 variables (age, vaccination status, pneumonia, stunting, shock, deworming, and daily weight gain per kilogram) were found to be independent predictors.

The aHR for nutritional recovery decreased by 1.9% for every 1-month increase in child age (aHR, 0.98; 95% CI, 0.97 to 0.99). Regarding vaccination status, under-5 children who were fully vaccinated for their age were 1.64 times more likely to recover than children who were not fully vaccinated for their age (aHR, 1.64; 95% CI, 1.20 to 2.24).

Regarding comorbid diseases, children who had pneumonia were 24.0% less likely to recover than those who did not (aHR, 0.76; 95% CI, 0.60 to 0.97) (Table 4).

## DISCUSSION

The aims of the present study were to determine recovery time from SAM in under-5 children and to identify predictors of nutritional recovery time.

Of the 423 children included in the study, 81.3% recovered, and the median nutritional recovery time of the entire cohort was 15.00 days. This recovery time falls within the acceptable maximum standard (< 28.00 days). However, approximately 13.0% of children with SAM stayed in the hospital beyond 28.00 days, and about 3.0% of children stayed beyond 42.00 days, which is an alarming length of hospitalization according to the Sphere standards.

The median nutritional recovery time was consistent with the median recovery time reported in studies performed in Bahir Dar, at Wolisso St. Luke Catholic Hospital, at Dilla University Referral Hospital, and in Zambia [18,21,30,36]. However, it was longer than reported nutritional recovery times in studies conducted at Dilchora Referral Hospital, in the North Shoa Zone of Ethiopia, in India, in Zambia, and in the Hadiya Zone of Ethiopia [17,19,24, 37,38]. The relatively short nutritional recovery times found in India and Zambia may be due to socioeconomic status, as well as treatment and caring practices, and the longer recovery time in the present study compared to other settings in Ethiopia might be because in this study setting, complicated cases may be referred, potentially prolonging the recovery time. In contrast, the nutritional recovery time in the present study was shorter than those found in studies conducted in Gambia, in the Gedeo Zone of Ethiopia, and at Ayder Referral Hospital [20,39,40].

Regarding predictors of nutritional recovery time, of all the sociodemographic characteristics examined in this study, age was the only factor to have a significant impact. For every 1-month increase in child age, the child was 1.9% less likely to recover than children at a comparatively younger age. This may be scientifically explained by the discontinuation of breastfeeding and the introduction of inappropriate complementary feeding practices as the age of the child increases. The present study is consistent with studies performed at the Debre Markos and Finote Selam hospitals and in northern India [22,24]. However, this study contrasts with studies conducted at Wolisso St. Luke Catholic Hospital, in the Gamo-Gofa Zone, and at the Shebedino *woreda* Outpatient Treatment Program Center [21,25,27,41]. This variation might have been due to differences in sample size and health care setups of the facilities.

Regarding immunization status, under-5 children who were fully vaccinated for their age were about 64% more likely to recover than children who were not fully vaccinated. The immune systems of unvaccinated children cannot fend off major childhood diseases such as malaria, pneumonia, diarrhea, and measles. Immunosuppression becomes more pronounced in starving children; as result, a child experiencing starvation will take longer to recover from those childhood diseases [42,43]. The present study is consistent with the findings of a study conducted in Bahir Dar [34]. Nevertheless, studies conducted in North Shoa as well as the *woreda* of Enderta in the Tigray Region showed no association between vaccination status and nutritional recovery time [19,25]. The reason for this variation could be differences in the health care system and sample size.

Among all comorbidities, diseases like pneumonia, stunting, and shock were the only significant predictors of recovery time from SAM. Relative to children who did not have pneumonia, children with pneumonia were 24.0% less likely to recover. This can be explained in terms of the synergistic relationship between pneumonia and malnutrition. The present study aligns with the results of retrospective cohort studies done in Zambia and at Debre Berhan Referral Hospital, Enat General Hospital, and Mehal Meda Primary Hospital [19,36,37]. However, pneumonia was not a significant predictor of nutritional recovery time in retrospective cohort studies performed in Southern Ethiopia, the Wolaita Zone, and Bahir Dar [18,26,44]. The reason for this difference may be that in those hospitals, pneumonia might be more likely to be detected and treated early than in the present study setting, since the present study setting is referral.

Similarly, children who were stunted were 33.0% less likely to recover than children who were not stunted. The explanation for this association may be that the management of acute malnutrition is similar regardless of whether stunting is present, although of course the most stunted children will have the highest risk of failure to respond to therapy and requiring a longer hospital stay.

Likewise, children who were in shock during treatment were 47.0% less likely to recover than children who were not. Unless prevented and detected early, shock can compromise many vital organs, including the brain, heart and kidneys, especially in children experiencing starvation of cellular energy [1]. However, shock was not a significant associated factor for recovery time from SAM in a study conducted in 2 hospitals in Wolaita [44]. The reason for this difference might be that in those hospitals, health care providers could facilitate prevention and early treatment of the underlying causes of shock, since the health care facilities were near the patients’ areas of residence. However, since the present study setting was referral-based, children from different regions of the country traveled for many hours to arrive at the study location. Hence, shock might not have been identified and treated as early, potentially resulting in prolonged hospitalization.

Regarding treatments, supplements and therapeutic feedings, deworming of children was the only significant factor associated with nutritional recovery time. Under-5 children who were not dewormed were 74.0% less likely to recover than those who were dewormed. A scientific explanation for this report could be that intestinal worms disrupt the nutrients available to children and prolong recovery time [1]. However, deworming was not associated with nutritional recovery time in studies done in the city of Bahir Dar, Shebedino, and southwest Ethiopia. This lack of an association may be explained by the smaller sample sizes of those studies and the fact that only small proportions of children were dewormed in the above studies [18,26,27].

Lastly, children with an average daily weight gain of ≥ 8 g/kg were 2.16 times more likely to recover than children whose average daily weight gain was < 8 g/kg. The scientific explanation for this association is clear; for marasmic children, a certain amount of daily weight gain is necessary to recover as fast as possible, since weight gain is one of the criteria for discharge. The present study is in line with a study done in the city of Bahir Dar [18], but other studies have not incorporated weight gain as a variable to be analyzed [19,22].

In general, performance indicators such as the recovery rate and nutritional recovery time were within the acceptable range. In the multivariate Cox proportional hazard regression analysis, a daily weight gain of ≥ 8 g/kg, full vaccination, and deworming were shown to reduce the nutritional recovery time. Conversely, older age, the presence of pneumonia, the presence of stunting, and the presence of shock were demonstrated to increase the nutritional recovery time.

To reduce the length of hospitalization, the Federal Ministry of Health should strengthen immunization and deworming programs and focus on early detection and treatment of SAM to prevent stunting. Additionally, the staff members of Yekatit 12 Hospital are strongly advised to perform daily weight gain monitoring and comply with national inpatient SAM management guidelines in the early diagnosis and treatment of pneumonia and shock. Lastly, future researchers are advised to use a prospective cohort study design to better reflect information including factors such as parental socio-demographic and socioeconomic characteristics, the educational status of health workers, and perceptions of caregivers regarding the term SAM.

## Figures and Tables

**Table 1.** Distribution of comorbid diseases among patients with severe acute malnutrition admitted to the therapeutic feeding unit of Yekatit 12 Hospital in Addis Ababa, Ethiopia from January 1, 2016 to December 30, 2018 (n=423)

Variables	Frequency (%)
HIV/AIDS	
Yes	39 (9.2)
No	384 (90.8)
Anemia	
Yes	181 (42.8)
No	242 (57.2)
Dehydration	
Yes	83 (19.6)
No	340 (80.4)
Fever	
Yes	140 (33.1)
No	283 (66.9)
Axillary temperature (°C)	
<38	62 (14.7)
≥38	79 (18.7)
Congenital heart disease	
Yes	40 (9.5)
No	383 (90.5)
Diarrheal disease(s)	
Yes	224 (53.0)
No	199 (47.0)
Tuberculosis	
Yes	24 (5.7)
No	399 (94.3)
Pneumonia	
Yes	179 (42.3)
No	244 (57.7)
Gastroenteritis	
Yes	202 (47.8)
No	221 (52.2)
Sepsis	
Yes	85 (20.1)
No	338 (79.9)
Rickets	
Yes	70 (16.5)
No	353 (83.5)
Stunting	
Yes	106 (25.1)
No	317 (74.9)
Global developmental delay	
Yes	42 (9.9)
No	381 (90.1)
Shock	
Yes	37 (8.7)
No	386 (91.3)
Microcephaly	
Yes	31 (7.3)
No	392 (92.7)

**Table 2.** Distribution of routine medications, special medications, supplements, and therapeutic feeding for patients with severe acute malnutrition cases admitted to the therapeutic feeding unit of Yekatit 12 Hospital in Addis Ababa, Ethiopia from January 1, 2016 to December 30, 2018 (n=423)

Variables	Frequency (%)
Routine treatments administered	
IV antibiotic(s)	
Yes	378 (89.4)
No	45 (10.6)
Oral antibiotic(s)	
Yes	205 (48.5)
No	218 (51.5)
Albendazole/mebendazole	
Yes	22 (5.2)
No	63 (14.9)
Not applicable	338 (79.9)
Special medication	
IV fluids	
Yes	67 (15.8)
No	356 (84.2)
ReSoMal	
Yes	180 (42.6)
No	243 (57.4)
Supplements administered	
Vitamin A	
Yes	69 (16.3)
No	354 (83.7)
Iron	
Yes	92 (21.7)
No	331 (78.3)
Folic acid	
Yes	160 (37.8)
No	263 (62.2)
Zinc	
Yes	61 (14.4)
No	362 (85.6)
Therapeutic foods administered	
Formula milk-75	
Yes	310 (73.3)
No	113 (26.7)
Formula milk-100	
Yes	390 (92.2)
No	33 (7.8)
RUTF	
Yes	145 (34.3)
No	278 (65.7)

IV, intravenous; ReSoMal, rehydration solution for malnutrition; RUTF, ready-to-use therapeutic food.

**Table 3.** Kaplan-Meier survival estimates for the recovery time from severe acute malnutrition according to different covariates at the therapeutic feeding unit of Yekatit 12 Hospital in Addis Ababa, Ethiopia from January 1, 2016 to December 30, 2018 (n=423)

Characteristics	Category	Median recovery time (d)				
		Estimate	95% CI		Log-rank	p-value^1^
			UL	LL	χ^2^ value	
Age (mo)	<24	15.00	13.73	16.27	6.739	0.009
	≥24	19.00	16.19	21.81		
Residence	Urban	14.00	12.66	15.34	17.401	<0.001
	Rural	20.00	17.70	22.30		
Type of malnutrition	Marasmus	14.00	12.68	15.32	14.769	0.001
	Kwashiorkor	21.00	18.45	23.55		
	Marasmic kwashiorkor	21.00	17.84	24.16		
Vaccination status	Fully vaccinated	13.00	11.68	14.32	34.031	<0.001
	Partially vaccinated	19.00	17.09	20.90		
	Not vaccinated	23.00	19.41	26.58		
HIV/AIDS	Yes	21.00	18.51	23.49	6.194	0.013
	No	15.00	13.76	16.23		
Anemia	Yes	17.00	14.79	19.21	5.860	0.015
	No	14.00	12.27	15.73		
Tuberculosis	Yes	21.00	14.09	27.91	5.678	0.017
	No	15.00	13.76	16.24		
Pneumonia	Yes	19.00	17.11	20.90	18.202	<0.001
	No	13.00	11.62	14.38		
Stunting	Yes	20.00	17.53	22.47	17.775	<0.001
	No	14.00	12.87	15.13		
Shock	Yes	26.00	20.55	31.45	13.094	<0.001
	No	15.00	13.76	16.24		
Deworming	Yes	9.00	7.16	10.84	23.228	<0.001
	No	23.00	19.95	26.04		
IV fluid	Yes	25.00	19.82	30.18	15.351	<0.001
	No	15.00	13.73	16.27		
ReSoMal	Yes	14.00	12.71	15.29	5.281	0.022
	No	17.00	15.17	18.83		
Daily weight gain (g/kg/d)	<8	20.00	18.83	21.17	79.509	<0.001
	≥8	11.00	10.01	11.99		
Overall		15.00	13.61	16.39		

CI, confidence interval; UL, upper limit; LL, lower limit; IV, intravenous; ReSoMal, rehydration solution for malnutrition.

1 A p-value of ≤0.05 indicates the presence of a significant difference between groups of predictor variables.

**Table 4.** Factors associated with recovery time from severe acute malnutrition among under-5 children at the therapeutic feeding unit of Yekatit 12 Hospital in Addis Ababa, Ethiopia (n=423)

Covariates	Category	cHR (95% CI)	aHR (95% CI)	p-value
Age		0.98 (0.98, 0.99)	0.98 (0.97, 1.00)	0.001
Sex	Male	1.23 (0.99, 1.53)	0.84 (0.67, 1.06)	0.062
	Female	1.00 (reference)	1.00 (reference)	
Residence	Urban	1.60 (1.27, 2.03)	1.20 (0.93, 1.56)	<0.001
	Rural	1.00 (reference)	1.00 (reference)	
Type of malnutrition	Marasmus	1.00 (reference)	1.00 (reference)	
	Kwashiorkor	0.63 (0.45, 0.85)	0.86 (0.41, 1.84)	0.002
	Marasmic kwashiorkor	0.64 (0.45, 0.91)	0.78 (0.36, 1.70)	0.014
Vaccination status	Fully vaccinated	2.14 (1.61, 2.86)	1.64 (1.20, 2.24)	<0.001
	Partially vaccinated	1.40 (0.96, 2.04)	1.25 (0.84, 1.87)	0.083
	Not vaccinated	1.00 (reference)	1.00 (reference)	
HIV/AIDS	Yes	0.65 (0.46, 0.93)	0.86 (0.59, 1.26)	0.018
	No	1.00 (reference)	1.00 (reference)	
Anemia	Yes	0.78 (0.63, 0.96)	1.08 (0.85, 1.36)	0.020
	No	1.00 (reference)	1.00 (reference)	
Dehydration	Yes	0.83 (0.63, 1.09)	1.08 (0.76, 1.54)	0.178
	No	1.00 (reference)	1.00 (reference)	
Fever	Yes	1.16 (0.92, 1.45)	1.07 (0.82, 1.40)	0.210
	No	1.00 (reference)	1.00 (reference)	
Diarrhea	Yes	0.84 (0.68, 1.04)	0.72 (0.46, 1.13)	0.110
	No	1.00 (reference)	1.00 (reference)	
Tuberculosis	Yes	0.58 (0.37, 0.93)	0.98 (0.59, 1.65)	0.024
	No	1.00 (reference)	1.00 (reference)	
Pneumonia	Yes	0.64 (0.51, 0.79)	0.76 (0.60, 0.97)	<0.001
	No	1.00 (reference)	1.00 (reference)	
Age	Yes	0.86 (0.70, 1.07)	1.08 (0.68, 1.71)	0.174
	No	1.00 (reference)	1.00 (reference)	
Rickets	Yes	0.77 (0.59, 1.02)	0.98 (0.72, 1.32)	0.071
	No	1.00 (reference)	1.00 (reference)	
Stunting	Yes	0.60 (0.47, 0.77)	0.67 (0.50, 0.88)	<0.001
	No	1.00 (reference)	1.00 (reference)	
Shock	Yes	0.48 (0.31, 0.73)	0.53 (0.32, 0.87)	0.001
	No	1.00 (reference)	1.00 (reference)	
Microcephaly	Yes	0.76 (0.49, 1.15)	0.84 (0.54, 1.31)	0.196
	No	1.00 (reference)	1.00 (reference)	
IV antibiotics	Yes	0.76 (0.53, 1.08)	0.96 (0.65, 1.41)	0.130
	No	1.00 (reference)	1.00 (reference)	
Deworming	Yes	1.00 (reference)	1.00 (reference)	<0.001
	No	0.33 (0.20, 0.55)	0.26 (0.11, 0.61)	
IV fluid	Yes	0.55 (0.40, 0.75)	0.79 (0.52, 1.21)	<0.001
	No	1.00 (reference)	1.00 (reference)	
ReSoMal	Yes	1.27 (1.03, 1.57)	1.20 (0.90, 1.59)	0.027
	No	1.00 (reference)	1.00 (reference)	
Vitamin A	Yes	1.22 (0.91, 1.64)	1.35 (0.97, 1.86)	0.173
	No	1.00 (reference)	1.00 (reference)	
Daily weight gain (g/kg/d)	<8	1.00 (reference)	1.00 (reference)	<0.001
	≥8	2.27 (1.76, 2.93)	2.16 (1.64, 2.84)	

Bivariate Cox regression analysis was done for each predictor variable. Then, variables that had p≤0.25 in the binary Cox regression analysis were entered into the multivariate Cox regression analysis. All variables had p≤0.25 in the binary Cox regression analysis. cHR, crude hazard ratio; CI, confidence interval; aHR, adjusted hazard ratio; AGE, acute gastroenteritis; IV, intravenous; ReSoMal, rehydration solution for malnutrition.
